# Single‑cell and spatial transcriptomics in chronic lung disease: cellular heterogeneity and molecular mechanisms in IPF and COPD

**DOI:** 10.1186/s12931-026-03836-7

**Published:** 2026-07-24

**Authors:** Xiaolan Huang, Fangfang Qu, Qun Luo, Yixue Li

**Affiliations:** 1 Zhongshan School of Medicine, Sun Yat-Sen University, Guangzhou, 510080 China; 2 Guangzhou National Laboratory, No. 9 XingDaoHuanBei Road, Guangzhou International Bio Island, Guangzhou, Guangdong Province 510005 China; 3 GMU-GIBH Joint School of Life Sciences, The Guangdong-Hong Kong-Macao Joint Laboratory for Cell Fate Regulation and Diseases, Guangzhou Medical University, Guangzhou, 511436 China; 4 State Key Laboratory of Respiratory Disease, National Clinical Research Center for Respiratory Disease, National Center for Respiratory Medicine, Department of Respiratory and Critical Care Medicine, Guangzhou Institute of Respiratory Health, The First Affiliated Hospital of Guangzhou Medical University, Guangzhou, Guangdong 510120 China

**Keywords:** Single-cell RNA sequencing, Spatial transcriptomics, Chronic lung diseases, COPD, IPF, Cellular heterogeneity, Pathogenesis, Therapeutic targets

## Abstract

Single-cell RNA sequencing (scRNA-seq) and spatial transcriptomics (ST) have advanced the study of complex lung diseases by resolving cellular heterogeneity, tissue architecture, and intercellular communication. These technologies are particularly informative in chronic respiratory disorders, where pathological remodeling involves coordinated alterations across epithelial, stromal, immune, and endothelial compartments. Idiopathic pulmonary fibrosis (IPF), a rare fatal fibrotic disorder, and chronic obstructive pulmonary disease (COPD), a prevalent heterogeneous respiratory condition, represent distinct chronic lung diseases shaped by persistent injury, maladaptive repair, and progressive tissue remodeling. However, current understanding of their pathogenesis remains incomplete, and conventional approaches lack the resolution to define disease-associated cell states, spatially restricted niches, or compartment-specific interactions. This review synthesizes recent scRNA-seq and ST studies in IPF and COPD, emphasizing how high-resolution atlases refine established pathobiological frameworks and reveal shared and disease-specific cellular programs. We highlight epithelial dysfunction and impaired progenitor repair as shared mechanistic features of IPF and COPD, while emphasizing alveolar regenerative failure, fibroblast activation, and profibrotic niche formation as central features of IPF, and compartment-specific airway remodeling, inflammatory epithelial states, and emphysema-associated parenchymal injury as prominent features of COPD. We further discuss stromal and immune remodeling, hypoxia-associated vascular remodeling, and the translational implications of these findings for molecular stratification, target prioritization, and mechanism-informed therapeutic development, while noting limitations related to end-stage tissue sampling, platform constraints, and mouse–human correspondence. This review provides an integrated framework for understanding the cellular and spatial mechanisms underlying the pathogenesis and translational relevance of IPF and COPD.

## Introduction

Chronic lung diseases commonly involve persistent epithelial injury, dysregulated repair, chronic inflammation and progressive remodeling of epithelial, stromal, and immune compartments. Among them, idiopathic pulmonary fibrosis (IPF) and chronic obstructive pulmonary disease (COPD) represent two clinically important and mechanistically informative disorders that affect distinct but overlapping compartments of the respiratory system and contribute substantially to the global burden of non-communicable diseases [1].

IPF is a rare, progressive fibrotic lung disease, and recent epidemiologic analyses suggest increasing incidence and prevalence in many regions despite substantial variation across case definitions and study settings [2, 3]. The disease is characterized by progressive scarring and distortion of the alveolar architecture, ultimately leading to respiratory failure [4], with a median survival of only 3–5 years after diagnosis [5]. Complex interactions among environmental triggers, including smoking and dust exposure, and genetic predisposition contribute to its pathogenesis [6–8]. Currently, nintedanib and pirfenidone are the two principal approved antifibrotic therapies for IPF [9]. Although these agents slow lung function decline, they do not reverse established fibrosis. Lung transplantation remains an option for selected patients, but only a minority are eligible [10], highlighting the need for mechanism-based therapeutic strategies.

COPD is a highly prevalent and heterogeneous disease characterized by incompletely reversible airflow limitation [11]. Pathologically, COPD involves two closely related but anatomically distinct compartments: small-airway inflammation and remodeling, including epithelial alterations and luminal narrowing, and progressive destruction of the distal alveolar parenchyma in emphysema [12]. Driven primarily by environmental exposures such as smoking and air pollution [13], COPD affects hundreds of millions of individuals worldwide [14]. Current treatments, including bronchodilators, anti-inflammatory strategies, and supportive interventions, primarily improve symptoms, reduce exacerbation risk, and support functional status, but have limited ability to reverse established structural damage or fundamentally modify long-term disease progression [15]. More effective mechanism-based therapies are therefore needed.

**Fig. 1:**
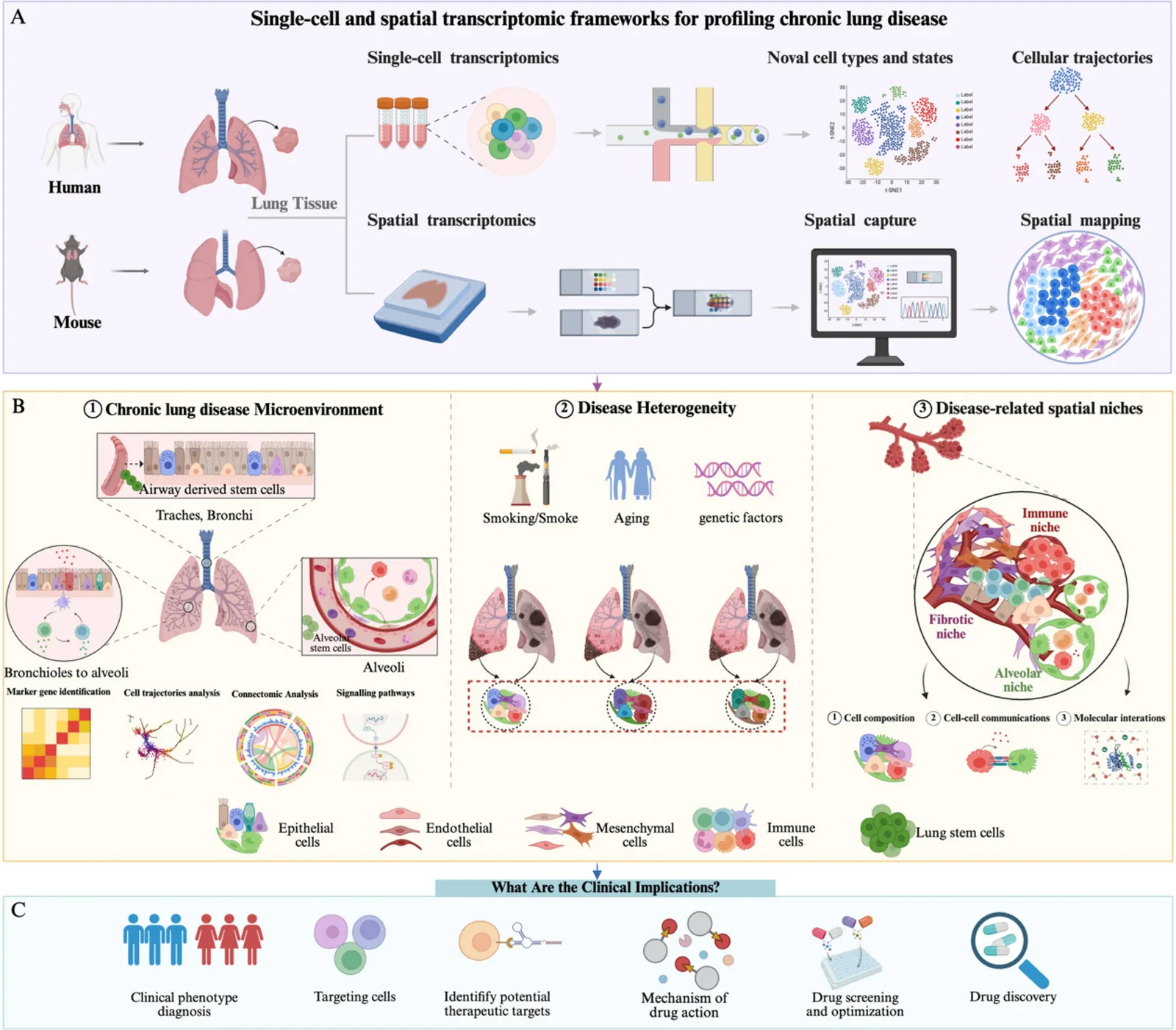
Integrative profiling of chronic lung diseases using single-cell and spatial transcriptomics. **A** Methodological frameworks including single-cell RNA sequencing (cell type/state identification and trajectory inference) and spatial transcriptomics (spatial niche characterization). **B** Key applications in chronic lung diseases: (1) Microenvironment profiling (e.g., marker gene identification, trajectory mapping); (2) Etiological heterogeneity (smoking, aging, genetic factors); (3) Disease-associated spatial niches (immune, fibrotic, alveolar) with composition and cellular crosstalk. **C** Clinical implications including phenotypic classification, cellular targeting strategies, and therapeutic development. Figure created in https://BioRender.com

Resolving the cellular states, lineage perturbations, and signaling niches that sustain IPF and COPD therefore requires approaches beyond conventional bulk or histological analyses. In this review, we synthesize how single-cell RNA sequencing (scRNA-seq) and spatial transcriptomics (ST) refine established pathobiological frameworks within a disease-centered structure while highlighting key points of convergence and divergence between the two diseases. Both diseases involve persistent epithelial injury, maladaptive repair, and multicompartment remodeling, but they diverge in their dominant pathological outcomes: IPF is marked by AT2 cell failure, fibroblast activation, and fibrotic niche formation, whereas COPD is characterized by small-airway remodeling, inflammatory epithelial reprogramming, and emphysematous destruction of the distal parenchyma. Accordingly, we focus on epithelial dysfunction, multicellular crosstalk, and the spatial organization of disease-associated niches, while also considering the translational implications and current limitations of these approaches (Fig. 1).

### From established pathology to single-cell and spatial insights in IPF and COPD

#### The established pathobiological frameworks of IPF and COPD

Before the development of high-resolution transcriptomic technologies, pathological, experimental, and clinical studies had established the core disease frameworks of IPF and COPD. In IPF, the prevailing model centers on repetitive alveolar epithelial injury, aberrant epithelial activation, failed regenerative repair, fibroblast and myofibroblast accumulation, and progressive extracellular matrix deposition, culminating in fibroblastic foci and irreversible fibrotic remodeling [16]. Immune activation, including macrophage-associated profibrotic signaling, has also been recognized as part of the fibrotic microenvironment, although its cellular organization has remained incompletely resolved [17].

Conversely, COPD has traditionally been viewed as involving interacting airway and distal parenchymal compartments, in which small-airway inflammation and remodeling contribute to airflow limitation, whereas alveolar wall destruction and loss of elastic recoil drive emphysema [18, 19]. Thus, both IPF and COPD involve epithelial injury and remodeling across lung compartments, but they differ in dominant anatomical distribution and pathological outcomes.

#### The resolution gap and the necessity of single-cell and spatial omics

Although these traditional frameworks have guided the field, conventional methodologies such as bulk RNA-seq and bulk tissue histology are limited by a substantial resolution gap. Bulk transcriptomic approaches measure average gene expression across mixed cell populations, thereby obscuring cellular heterogeneity, masking rare or transitional states, and failing to resolve the local tissue context in which pathogenic interactions occur.

Single-cell RNA sequencing enables transcriptome-wide profiling at single-cell resolution and reveals cellular heterogeneity, dynamic cell states, rare subpopulations, and microenvironment-associated interaction programs [20]. In lung biology, scRNA-seq has expanded maps of cellular diversity, with large-scale studies profiling millions of cells and identifying previously unrecognized cell states across health and disease [21–23]. However, because tissue dissociation disrupts anatomical structure, scRNA-seq alone has limited capacity to localize these states or define their organization within pathological niches.

Spatial transcriptomics addresses this limitation by mapping RNA expression onto preserved histological architecture [24], enabling the localization of pathogenic cell states and their surrounding microenvironments. The technical principles, platform-specific features, and analytical workflows of ST have been reviewed elsewhere [25–27]. Here, we focus on the biological contribution of ST by reconnecting transcriptionally defined cell states with anatomical compartments, pathological lesions, and disease-associated niches. In this context, integrated scRNA-seq and ST approaches help clarify which epithelial, stromal, and immune states underlie known lesions, how profibrotic niches are organized in IPF, and how disease-associated states are distributed across the small airways and distal parenchyma in COPD. Recent studies have therefore begun to refine, rather than replace, traditional pathobiological models of IPF and COPD [28, 29] (Tables 1 and 2).

**Table 1:** Disease-associated cell states in IPF identified by single-cell and spatial transcriptomic studies

Cell Type/State	Characteristic Features	Markers	Molecules/Pathways	Species	Ref
Epithelial and progenitor cell states					
SPB	Basal cells primed for secretory differentiation	KRT5, KRT17, CEACAM6, SERPINs	O-glycan biosynthesis; Notch3 signaling	Human	[30]
Metaplastic KRT5 + basal cells	Metaplastic epithelial stem cell	KRT5, TP63, KRT17	Drive alveolar remodeling in fibrosis; TGFβ1 signaling	Human	[31]
Aberrant Basaloid cells	Pathologic epithelial cells enriched in fibrotic regions with mesenchymal-like and matrix-associated features	TP63, KRT17, KRT8, LAMB3, LAMC2, VIM, CDH2, FN1, COL1A1, MMP7,	EMT-associated programs; senescence-related genes; TGF-β activation; integrin signaling; SOX9	Human	[28, 32]
ABIs	Transitional cell	KRT8, KRT17	Intermediates in hAEC2 to basal transdifferentiation; TGFβ1 signaling; BMP signaling	Human	[31]
Krt8 + ADI	Transitional stem cell state	Krt8, Cldn4, Areg, cdkn1α, S100a6	p53 and NFκB activation; Cellular senescence; Epithelial-mesenchymal transition	mouse; Human	[33]
DATPs	Transitional epithelial progenitors derived from AT2 cells	Cldn4, Krt8, Ndrg1, Sprr1a, AW112010	p53 signaling; hypoxia response (Hif1α); glycolysis; Epithelial-mesenchymal transition	mouse; Human	[34]
PATS	Transitional epithelial progenitors derived from AEC2s, intermediate between AEC2s and AEC1s	Cldn4, Krt19, Sfn,, Ctgf, Lgals3, Sox4	p53 signaling; TGFβ signaling; DNA-damage response; Cellular senescence; Cytoskeletal remodeling	mouse	[35]
PATS-Like cells	aberrant transitional epithelial cells in fibrotic foci	SFN, KRT17, TP63, CLDN4	p53 signaling; DNA-damage response; cellular senescence; TGFβ signaling	Human	[35]
Cdc42-null AT2	Cdc42—deficient alveolar stem cells with impaired differentiation capacity	Sftpc, Cdc42 deficient	TGF-β signaling activation; Cytoskeleton remodeling; Focal adhesion	mouse	[36]
LNEPs	H2-K1 high progenitors with multiple cell regeneration	H2-K1, AW112010, Cd74, Cd14	Host-defense signaling pathways; Interferon-regulated genes	mouse	[37]
Profibrotic stem cell	Profibrotic basal stem cell-like variant	TP63, KRT17, CEACAM6, VIM, CXCL17, IL1RN, CLDN4	Myofibroblast-activating programs; EGFR, mTOR, WNT, and TGF-β signaling	Human; mouse	[38]
Fibroblasts and stromal cell states					
Meflin + fibroblasts	Proliferating fibroblast population with anti-fibrotic properties	ISLR (Meflin)	Inhibition of TGF-β-induced fibrogenesis; ECM homeostasis regulation	Human; mouse	[39]
CTHRC1 + fibroblasts	Pathologic collagen-producing fibroblasts contributing to fibrotic niches	CTHRC1, TGFB, SPP1, COL1A1, COL3A1, TNC, POSTN	TGFβ1 signaling; Excessive ECM production; migration	Human; mouse	[31, 40, 41]
HAS1 + fibroblasts	Subpleural ECM-producing fibroblasts	LUM, PDGFRA, COL1A1, HAS1, HAS2**,** TWIST	IL4/IL13 signaling; TWIST, SNAI1, ECM remodeling; BMP antagonism	Human	[28, 31]
ACTA2 + Myofibroblasts	Activated fibroblasts in subepithelial regions	ACTA2, LUM, COL1A1	ECM expansion; TGF-β signaling	Human	[28]
PLIN2 + lipofibroblast-like	Lipid-associated fibroblasts in interstitial regions	PLIN2, LUM, PDGFRA	Lipid metabolism; Diffuse ECM deposition	Human	[28]
AF1	Mesenchymal progenitor cell	PDGFRA, LIMCH1, TCF21	Notch signaling; Wnt/Fgf signaling	Human; mouse	[42]
AF2	Notch-dependent niche cell	PDGFRB, COX4I2, NOTCH3	Notch signaling pathway	Human; mouse	[42]
Immune cell states					
SPP1^hi^ Macrophages	Profibrotic interstitial macrophages with increased proliferation	SPP1, MERTK, LGMN, SIGLEC10	Stress response; extracellular matrix organization; fibroblast activation	Human	[43]
Profibrotic macrophages	Profibrotic, monocyte-derived macrophages associated with fibrosis	SPP1, CHI3L1, IL1RN, MMP9, CSF1, SPARC, GPCA, PALLD, CTSK	Exocytosis, secretion; Cholesterol ester metabolism (LIPA); ECM degradation (MMP9); CSF1 autocrine loop	Human	[32, 44]
IPFeMΦ	Transitional macrophage subset with hybrid alveolar-interstitial features, pro-fibrotic signature, M2-like polarity	SPP1, CD84, CD36, CD9, APOE, CCL18, GPNMB	ECM remodeling; IL-6 signaling; PDGF signaling; lipid metabolism	Human	[45]
Transitional profibrotic macrophages	Monocyte-derived macrophages with transitional profibrotic gene expression programs	Cx3cr1, Ccr2, Mafb, MHCII, Pdgfa	PDGF-AA-mediated fibroblast proliferation; M-CSF/M-CSFR signaling	mouse; Human	[46]
Monocyte—derived alveolar macrophages	Pro-fibrotic macrophages derived from recruited monocytes, localized to fibrotic niches	Mrc1, Pdgfa, Cdh1, Fn1, Mmp12, Mmp14, Spp1, Timp2, Csf1r, Csf1	M-CSF/M-CSFR signaling; PDGFA-mediated fibroblast proliferation	mouse; Human	[47]
Endothelial cell-associated states					
COL15A1 + VE cells	Ectopic peribronchial endothelial cells in distal fibrotic regions	COL15A1, PECAM1	Aberrant vascular remodeling;	Human	[32]
ACKR1 + venous ECs	Inflammatory and angiogenic venous ECs enriched in fibrotic lungs	ACKR1, SELP, TIFA	Inflammatory signaling; venous remodeling	mouse;Human	[48]
TrkB + Capillary ECs	Persistent activated capillary EC state associated with disrupted vascular homeostasis	NTRK2 (TrkB), FXDY5, SPP1, ANKRD37	TrkB/YAP/TAZ signaling; glycolysis; hypoxia-associated response; BDNF-TrkB crosstalk	mouse;Human	[48]

*Abbreviations: SPB* secretory primed basal, *RAS* Respiratory airway secretory, *ABIs* Alveolar-basal intermediates, *ADI* Krt8 + alveolar differentiation intermediate, *DATPs* Damage-associated transient progenitors, *PATS* pre-alveolar transitional state, *LNEPs* Club-like lineage-negative epithelial progenitors, *AF1/2* alveolar fibroblasts 1/2, *IPFeMΦ* IPF-expanded macrophages, *VE* vascular endothelial, *ECs* endothelial cells

**Table 2:** Disease-associated cell states in COPD identified by single-cell and spatial transcriptomic studies

Cell Type/State	Characteristic Features	Markers	Molecules/Pathways	Species	Ref
Epithelial and progenitor cell states					
AT2B	AT2 subpopulation with aberrant metabolism and reduced stress tolerance	SFTPC, SFTPA1, SFTPA2, ETV5, HHIP, CA2, WIF1	Metabolic and biosynthetic processes; antioxidant response; reduced stress tolerance associated with NUPR1 downregulation	Human; mouse	[29]
RAS	Secretory cells in respiratory bronchioles; Progenitors for AT2 cells	SCGB1A1, SCGB3A, CEACAM6, LAMP3	Altered transcriptome in COPD; Notch signaling involved in maintenance	Human	[49]
SCGB3A2 + AT2	Aberrant AT2 cell subpopulation; Hybrid state co-expressing secretory and AT2 markers	SCGB3A2, LAMP3, SFTPC	Abnormal states in COPD; Potential differentiation defect; Smoking-related	Human	[49]
CD66c⁺ AT2	IFN-sensitive subset of human alveolar stem cells	CD66c, CEACAM6	Lost in emphysema; IFNγ signaling; Wnt signaling	Human	[50]
asATII Cells	Emphysema—specific ATII in enlarged distal alveolar sacs	SPC, SCGB3A2, CD74	Inflammatory response; oxidative stress; Wnt signaling inhibition; senescence; mitochondrial dysfunction	Human	[51]
TASCs	Unique epithelial cells in distal airways, enriched in TBs	SCGB3A2, SFTPB, RNASE1, SFTPA2, HOPX, SFTPA1	Mediating Airway Transition; Reduced in COPD	Human	[52]
DASCs	Self-renewing distal airway progenitors	Krt5, p63	Regenerative therapy in COPD; Differentiation into AT1 cells; Notch and Wnt signalling	Human; mouse	[53]
GCM	COPD goblet metaplasia variant progenitor	p63, KRT5, TRPC6, SPC24, B4GALT1, EPPK1, TRA2A	Mucus hypersecretion; Airway obstruction	Human	[54]
SCM	COPD squamous cell metaplasia variant progenitor	p63, KRT5, SCD, FDPS, IDI1, SQLE	Squamous metaplasia, fibrosis promotion; TGF-beta signaling	Human	[54]
iSCM	COPD Inflammatory squamous cell metaplasia variant progenitor	p63, KRT5, CCL20, CXCL1, CXCL18, PLAU	Inflammatory signaling (CXCL8, IL-1β), neutrophil infiltration; Drives fibrotic responses	Human	[54]
Fibroblasts and stromal cell states					
Gli1 + fibroblasts	A stromal cell subset enriched around larger airways and vessels within the lung adventitia	Gli1, Il-7	Provide niche for TRLs; Hedgehog-IL-7 axis	mouse	[50]
Immune cell states					
TRLs	Lung-resident lymphocytes increased in emphysema	CD3, CD69, CD44, SELL-	Inhibit alveolar stem cell renewal; Respond to viral pathogens IFNγ; IL-7 signaling pathway; Th1 pathway	Human; mouse	[50]
High-MT macrophages	Alveolar macrophage subpopulation with high metallothionein expression	MT1G, MT1X, MT1E, MT2A, HMOX1	Enriched in advanced COPD; Antioxidant response; Metal ion sequestration	Human	[29]
IMCHIT1 macrophages	Interstitial profibrotic macrophages driving remodeling	CHIT1, CHI3L1, SPP1, MMP9	Associated with FEV1 decline and highly inflamed cellular communities	Human	[55]
Endothelial cell-associated states					
Aerocyte/aCap endothelial cells	Capillary endothelial subset associated with gas exchange and leukocyte migration	CA4, HPGD	Inflammatory markers (TNFRSF10D, IRF1) ↑	Human	[29]
gCap endothelial cells	Inflamed general capillary endothelial cells	FCN3, NOSTRIN	CXCL-associated signaling (CXCL12); inflammatory markers (TNFAIP3, IFI6) ↑	Human, Mouse	[29]
Senescence-associated ECs	IL6-high endothelial cells with senescence-associated programs in severe COPD	IL6, CDKN1A	Endothelial senescence; IL6-associated T-cell modulation	Human	[56]

*Abbreviations: RAS* Respiratory airway secretory, *TASC* Terminal airway-enriched secretory cells, *TBs* terminal bronchioles, *DASCs* Distal airway stem cell, *GCM* goblet cell metaplasia, *SCM* squamous cell metaplasia, *iSCM* inflammatory SCM, *TRLs* tissue-resident lymphocytes, *High-MT macrophages* high metallothionein macrophages, *aCap* alveolar capillary, *gCap* general capillary

### Refining IPF pathogenesis with single-cell and spatial transcriptomics

**Fig. 2:**
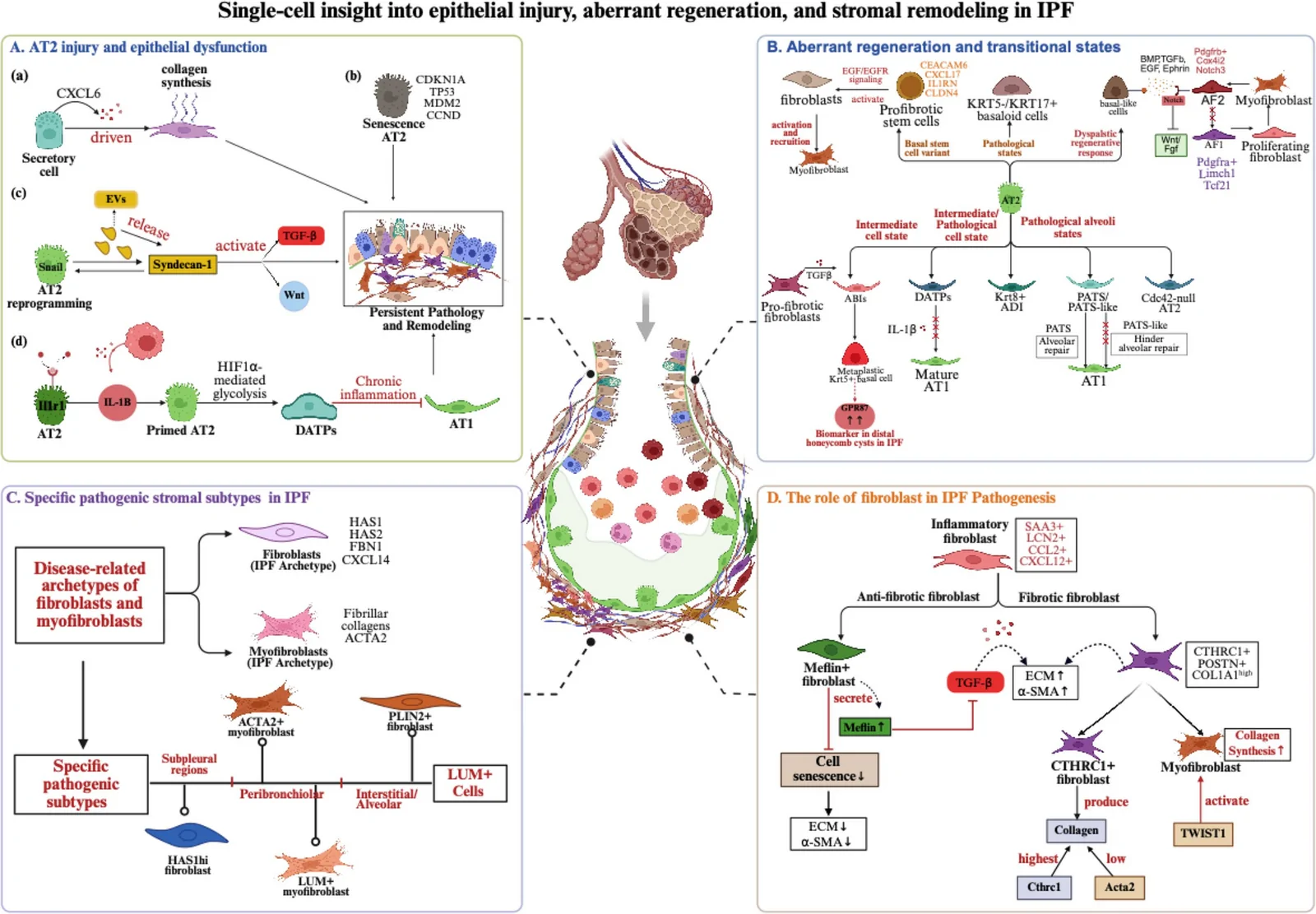
Single-cell insights into epithelial injury, aberrant regeneration, and stromal remodeling in IPF. **A** AT2 injury and epithelial dysfunction in IPF, including epithelial stress responses, AT2 reprogramming, impaired AT2-to-AT1 repair, and persistent pathological remodeling. **B** Aberrant regeneration and transitional states in IPF, highlighting intermediate epithelial populations, abnormal basaloid-like programs, and defective alveolar repair trajectories. **C** Specific pathogenic fibroblast and myofibroblast subtypes identified in IPF. **D** The role of fibroblasts in IPF pathogenesis, including fibroblast activation, TGF-β-driven profibrotic transition, myofibroblast differentiation, and extracellular matrix deposition. Abbreviations: AT1, alveolar epithelial type 1; AT2, alveolar epithelial type 2; ABI, Alveolar-basal intermediate; ADI, Krt8 + alveolar differentiation intermediate; DATPs, Damage-associated transient progenitors; PATS, pre-alveolar transitional state; AF1/2, alveolar fibroblasts1/2; EVs, Extracellular vesicles; HIF-1a, Hypoxia-inducible factor 1; ECM, Extracellular matrix; TGF-β, transforming growth factor-β. Figure created in https://BioRender.com

**Fig. 3:**
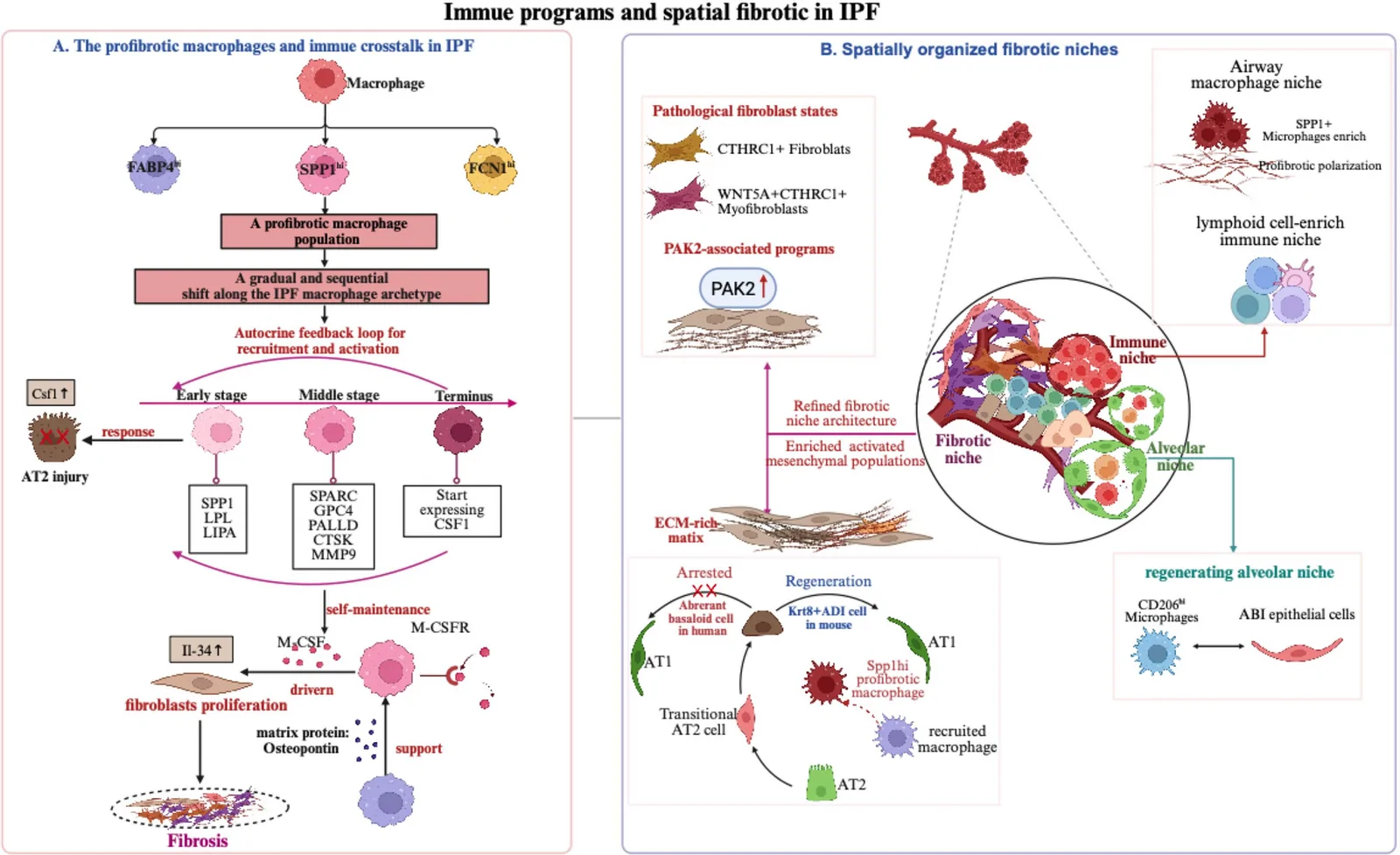
Immune programs and spatial fibrotic niches in IPF. **A** Profibrotic macrophage populations and immune crosstalk in IPF, including recruitment, activation, and macrophage-fibroblast interactions that promote fibrosis. **B** Spatially organized fibrotic niches in IPF, illustrating pathological fibroblast states, immune-enriched niches, macrophage-associated airway niches, and regenerating alveolar niches revealed by spatial transcriptomic analyses. Abbreviations: AT1, alveolar epithelial type 1; AT2, alveolar epithelial type 2; ADI, Krt8 + alveolar differentiation intermediate; ABI, Alveolar-basal intermediate; ECM, Extracellular matrix; IPF, idiopathic pulmonary fibrosis. Figure created in https://BioRender.com

**Fig. 4:**
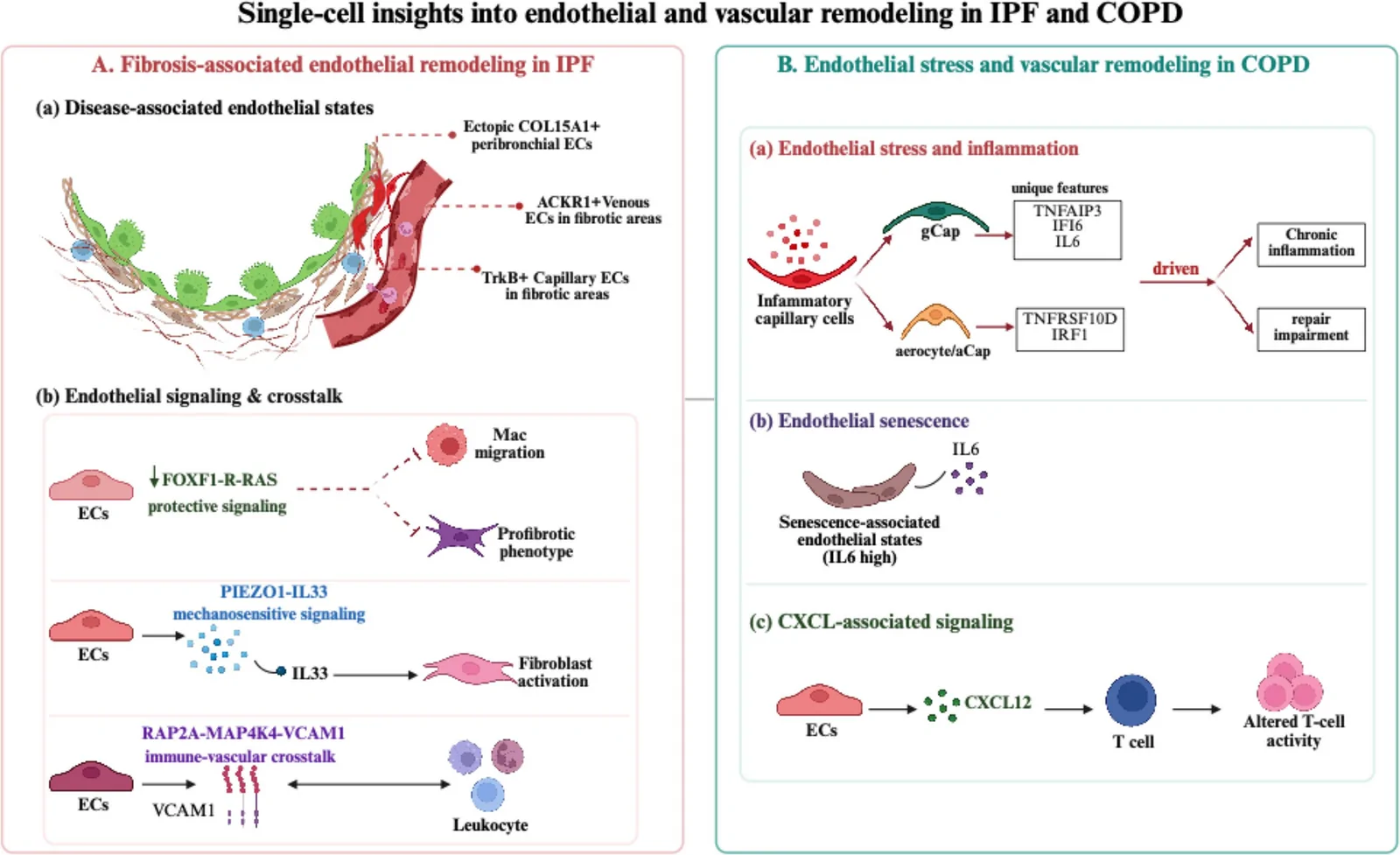
Single-cell insights into endothelial and vascular remodeling in IPF and COPD. **A** Fibrosis-associated endothelial remodeling in IPF, including ectopic COL15A1 + peribronchial endothelial cells, ACKR1 + venous endothelial cells, TrkB + capillary endothelial cells, and endothelial signaling programs involving FOXF1-R-Ras, PIEZO1-IL33, and RAP2A-MAP4K4-VCAM1. **B** Endothelial stress and vascular remodeling in COPD, including inflammatory capillary endothelial programs, altered gCap and aerocyte/aCap features, IL6-high senescence-associated endothelial states, and CXCL12-associated endothelial-T cell signaling. Abbreviations: IPF, idiopathic pulmonary fibrosis; COPD, chronic obstructive pulmonary disease; ECs, endothelial cells; gCap, general capillary; aCap, alveolar capillary; Mac, macrophage; VCAM1, vascular cell adhesion molecule 1. Figure created in https://BioRender.com

Single-cell and spatial transcriptomic studies have refined IPF pathogenesis by resolving epithelial, stromal, immune, and endothelial states within profibrotic tissue niches. The following sections examine these advances across epithelial injury and repair, stromal remodeling, immune crosstalk, endothelial remodeling, and spatially organized fibrotic niches (Table 1) (Figs. 2, 3 and 4).

#### Epithelial injury and aberrant repair

Repetitive injury to the aging alveolar epithelium is a central feature of IPF pathogenesis, and scRNA-seq studies have refined this concept by showing that epithelial damage is coupled to persistent defects in alveolar regeneration rather than effective tissue restoration [57]. In particular, alveolar type 2 (AT2) cells, the principal stem/progenitor population of the distal lung, display marked abnormalities in IPF, including altered stress responses, impaired differentiation capacity, and the depletion of normal reparative programs [44, 58]. These abnormalities indicate sustained dysfunction of the regenerative epithelial compartment.

Single-cell profiling has shown disruption of AT2-to-AT1 differentiation, which is associated with the accumulation of abnormal transitional epithelial states [31, 33]. In murine injury models, these populations emerge as transient repair intermediates, described as Krt8 + alveolar differentiation intermediates (ADIs) [33], damage-associated transient progenitors (DATPs) [34], and pre-alveolar type 1 transitional states (PATs) [35]. Mechanistically, Cdc42-deficient AT2 cells fail to complete AT1 differentiation and epithelial regeneration, while increased mechanical tension and TGF-β signaling can promote maladaptive repair programs and fibrotic remodeling [36]. Crucially, related aberrant basaloid [32] and KRT5-/KRT17 + pathological epithelial states [28] have also been identified in human IPF, with stress-associated, senescence-associated, and developmentally reactivated transcriptional programs. However, unlike the resolving intermediate states in mice, these populations in human IPF are thought to become pathologically prolonged or arrested, consistent with chronic failed regeneration [31]. Together, these observations identify impaired AT2 stem cell function and incomplete lineage progression as central features of maladaptive epithelial repair in IPF. This regenerative failure may also be metabolically constrained, as reduced fatty acid oxidation programs, including reduced CPT1a activity, are associated with mitochondrial dysfunction, abnormal intermediate states, and increased susceptibility to fibrotic remodeling [59]. Single-cell and spatial analyses have also implicated AT2 cells in homocysteine metabolism dysregulation during fibrosis, with reduced expression of the methionine-cycle enzyme MTRR linked to impaired STX17-dependent autophagic flux and increased fibrotic injury [60].

A second major theme is the redirection of distal epithelial repair toward basal-like reprogramming. Basal cells are normally proximal airway epithelial progenitors that maintain epithelial homeostasis and support repair after injury [61]. In IPF, expansion of basal or basal-like epithelial populations, together with bronchiolization of distal fibrotic regions, is a well-recognized feature of aberrant epithelial remodeling [62]. Early scRNA-seq studies identified epithelial states in IPF that are absent or rare in healthy lungs, including airway-associated basal-like, goblet-like, and atypical transitional populations [44, 57]. Subsequent analyses further showed that epithelial remodeling in IPF involves disruption of normal proximal–distal differentiation programs, with epithelial cells acquiring mixed or multilineage features not seen in the healthy lung [58]. Further single-cell analyses resolved basal cell heterogeneity in normal and IPF lungs, identifying multipotent progenitor basal cells (MPBs), proliferating basal cells (PBs), secretory-primed basal cells (SPBs), and activated basal cells (ABs), with SPBs particularly enriched in end-stage honeycomb lesions [30]. These findings suggest that IPF-associated basal cell programs comprise distinct epithelial states associated with activation, secretory priming, and pathological remodeling.

Within this altered repair landscape, human AT2 cells have been shown to undergo alveolar-to-basal reprogramming under profibrotic mesenchymal signaling, either directly or through alveolar-basal intermediate states (ABIs) [31]. Epithelial syndecan-1 overexpression may contribute to this maladaptive reprogramming by promoting TGF-β and Wnt signaling and altering extracellular vesicle cargo through reduced antifibrotic microRNA packaging [63]. This process may link depletion of functional AT2 cells to basal cell metaplasia in distal lung regions. Consistent with this view, large-scale single-cell studies identified an IPF-enriched population of aberrant basaloid cells, variably described as KRT5 −/KRT17 + pathological epithelial cells or aberrant basaloid cells [28, 32]. These cells express TP63 and KRT17, but lack canonical airway basal markers such as KRT5 and KRT15, and additionally co-express senescence markers, extracellular matrix genes, EMT-related programs, and established IPF-associated factors including MMP7 and integrin αVβ6 [28, 32, 64]. Their enrichment in honeycomb cysts and regions adjacent to myofibroblast foci supports their identity as a pathological epithelial state linked to active fibrotic remodeling [65]. Pseudotime analyses further suggest that these populations arise, at least in part, from transitional alveolar epithelial states [66], placing aberrant basaloid cells along a continuum from failed alveolar repair to maladaptive epithelial reprogramming. Hypoxia-inducible factors (HIFs) are oxygen-sensitive transcriptional regulators that coordinate cellular adaptation to hypoxia and are also influenced by inflammatory, redox, and metabolic states [67]. HIF-1α and HIF-2α have overlapping but context-dependent roles in hypoxia-associated metabolic adaptation, inflammatory responses, and tissue remodeling. In the lung epithelium, local hypoxia have been shown to influence epithelial fate decisions during alveolar regeneration [68]. In an acute injury context, scRNA-seq, lineage tracing, and organoid studies showed that IL-1β-driven HIF-1α-mediated glycolysis promotes AT2-to-DATP conversion and is required for subsequent AT1 differentiation, whereas sustained inflammatory signaling impairs DATP maturation and alveolar regeneration [34]. In contrast, recent work in repetitive injury models further implicates sustained HIF-2α signaling in maladaptive repair, linking airway-derived progenitors to the emergence of abnormal epithelial populations and showing that genetic or pharmacologic HIF-2α inhibition reduced aberrant epithelial state accumulation while improving alveolar repair [69]. Additional single-cell cloning studies have identified profibrotic basal stem cell variants in IPF that activate myofibroblasts and express fibro-inflammatory programs, further indicating that abnormal basal-like progenitor states may actively contribute to fibrogenesis rather than merely reflect epithelial injury [38].

Single-cell studies of lung progenitor populations have also expanded the spectrum of epithelial cell states implicated in alveolar repair failure. In murine bleomycin models, rare injury-responsive lineage-negative epithelial progenitors (LNEPs), marked by H2-K1, have been implicated in alveolar regeneration, but dysfunctional progenitor responses are associated with senescence, reduced self-renewal, and impaired restoration of alveolar structure [37]. In parallel, mesenchymal niche populations, including alveolar fibroblast subsets termed AF1 and AF2 cells, have been shown to form a Notch-dependent microenvironment with Krt5 + basal cells while suppressing Wnt/Fgf-associated regenerative signaling, thereby favoring maladaptive epithelial remodeling over effective AT2-mediated repair [42]. Together, these studies suggest that aberrant epithelial repair in IPF reflects intrinsic defects in AT2 and transitional epithelial states as well as altered progenitor regulation by the surrounding injury-associated niche.

Single-cell analyses support a higher-resolution model of IPF epithelial pathogenesis in which repetitive alveolar injury is coupled to failed regeneration, persistence of transitional epithelial states, and progressive basal-like reprogramming. AT2 dysfunction, abnormal progenitor activation, and emergence of aberrant basaloid populations together outline a pathological continuum linking epithelial injury to bronchiolization and fibrotic remodeling (Fig. 2A-B). Although epithelial stress and progenitor dysfunction are also evident in COPD, IPF epithelial pathology is more tightly linked to alveolar regenerative failure and profibrotic niche formation.

#### Fibroblast heterogeneity, matrix remodeling, and profibrotic crosstalk

Fibroblasts are central effectors of tissue remodeling in IPF, but single-cell studies have shown that the mesenchymal compartment is far more heterogeneous than suggested by traditional histologic definitions of “fibroblasts” and “myofibroblasts” (Fig. 2C-D) [70, 71]. These studies further indicate that matrix remodeling in IPF extends beyond collagen deposition, with distinct fibroblast states associated with ECM production, inflammatory signaling, stress responses, and niche remodeling [72, 73].

Initial single-cell analyses of human lung stromal cells identified separable fibroblast and myofibroblast populations based on distinct transcriptional signatures. Fibroblasts are characterized by the expression of genes such as CD34, FBN1, FBLN2, and VIT, whereas myofibroblasts express contractile and mechanoadhesive gene programs, including MYLK, NEBL, MYO10, and ITGA8 [32]. In IPF, these populations acquire disease-associated features, including collagen-rich and ACTA2-enriched myofibroblast programs, as well as fibroblast populations with increased HAS1, SHH-associated signaling, and CXCL14 expression [32]. Regional heterogeneity is also evident, with HAS1^hi^ fibroblasts localized to subpleural areas and PLIN2 + or LUM + subsets in the parenchyma [28]. These findings indicate that fibroblast activation in IPF is regionally patterned and cannot be captured by any single conventional marker. Accordingly, ACTA2, although widely used to define myofibroblasts, does not reliably distinguish pathological fibrosis-associated fibroblasts from other activated stromal cells [40, 74–76]. Single-cell analyses instead identify CTHRC1 as a relatively more specific marker of disease-associated profibrotic fibroblasts [40, 41]. In a murine fibrosis study, Tsukui et al. mapped collagen-producing mesenchymal cells and identified a distinct Cthrc1 + fibroblast population in alveolar regions with high ECM-producing activity [40]. Corresponding CTHRC1 + fibroblast states have also been recognized in human IPF datasets [41]. More recent integrated single-cell and spatial analyses further suggest that CTHRC1 + pathological fibroblast states are heterogeneous, including a WNT5A + CTHRC1 + myofibroblast population enriched in IPF and positioned near the terminal end of a pathological fibroblast differentiation trajectory [77]. Although COL1A1 remains a hallmark of this population, pathological fibroblast states express broader ECM-associated programs extending beyond fibrillar collagens to include FN1, TNC, POSTN, and COL3A1 [40, 74]. Fibrotic injury also increases ECM-related gene expression across multiple mesenchymal subtypes rather than exclusively within one terminal myofibroblast population, shifting the view of fibrosis from a general histologic picture of collagen accumulation toward distinct disease-associated stromal programs with specific matrix outputs [64, 78]. Mechanistically, single-cell-guided work has implicated SFRP2 as an autocrine amplifier of fibrogenic myofibroblast activity in IPF, linking SFRP2-high myofibroblasts to impaired PINK1-mediated mitophagy and sustained ECM production [79]. Trajectory analyses further suggest that these pathological states arise from resident alveolar fibroblasts, consistent with a transition from a homeostatic mesenchymal compartment to fibrosis-driving cell states [40].

Lineage-resolved studies in murine fibrosis models further suggest that alveolar fibroblasts can act as plastic progenitors of multiple injury-associated mesenchymal states. After injury, Scube2-labeled alveolar fibroblasts differentiate into Cthrc1 + Col1a1 + fibrotic fibroblasts, inflammatory fibroblasts enriched for chemokines such as Cxcl12 and Lcn2, stress-activated fibroblasts marked by Cdkn1a-associated programs, and proliferative fibroblasts [41]. Notably, ECM-associated programs are not entirely confined to mesenchymal cells, as prior single-cell studies also identified KRT5 −/KRT17 + pathological epithelial cells expressing COL1A1, FN1, and other pathologic ECM components [28, 32]. This finding supports the concept that fibrotic matrix remodeling is embedded within broader maladaptive repair programs rather than representing a purely stromal endpoint. Within the alveolar fibroblast compartment, some homeostatic fibroblast states exhibit lipogenic or lipofibroblast-like features, overlap in part with populations described as lipid-associated fibroblasts (LAFs), and are thought to support the alveolar epithelial niche [80, 81]. Mouse and human single-cell studies have identified lipofibroblast-associated programs marked by *TCF21, PLIN2, FGF10,* and additional lipogenic signatures, although marker definitions vary across datasets [71, 82]. In fibrosis, these homeostatic lipogenic programs are reduced or attenuated, whereas inflammatory and ECM-producing fibroblast states become more prominent, suggesting a shift from epithelial-supportive mesenchyme toward pathological remodeling [81]. Consistent with this, lineage-tracing and single-cell analyses in young mice suggested a reversible relationship between lipofibroblast-like states and Cthrc1 + activated myofibroblasts during fibrosis progression and resolution [83]. Because alveolar fibroblasts normally provide structural and metabolic support to the alveolar epithelium, these transitions underscore the coupling between epithelial injury and fibroblast-state remodeling during fibrosis [42].

Mechanistic signaling networks further connect fibroblast plasticity to multicellular crosstalk. In murine injury models, a Pdgfrb^hi^ fibroblast population defined by Notch3 and Nrarp regulates fibrogenesis through Notch-Wnt crosstalk [82, 84]. Related studies further suggest that Notch3-dependent differentiation from Pdgfra + alveolar fibroblasts (AF1) to Pdgfrb + alveolar fibroblasts (AF2), through an Acta2 + myofibroblast intermediate, contributes to formation of a pathological mesenchymal niche associated with aberrant epithelial repair [42]. Notably, not all fibroblast states appear uniformly profibrotic. Single-cell analyses have identified a Meflin + fibroblast population with antifibrotic properties, at least in part through antagonism of TGF-β-driven fibrogenesis, whereas loss of this program accompanies progressive fibrosis [39]. Integrative epigenomic analyses have further implicated regulatory factors such as TWIST1 in promoting pathological matrix programs and fibroblast activation [85].

Collectively, single-cell studies support a model in which lung-resident fibroblast populations, particularly alveolar fibroblasts, undergo injury-induced remodeling into a spectrum of pathological mesenchymal states in IPF. The emergence of CTHRC1 + fibrotic fibroblasts, the persistence of inflammatory and niche-forming fibroblast programs, and the loss of lipid-associated and Meflin + protective mesenchymal states together contribute to the progressive matrix remodeling and profibrotic crosstalk that characterize IPF (Fig. 2C-D).

#### Profibrotic macrophage programs and immune crosstalk

Single-cell transcriptomic profiling has refined the understanding of the immune landscape in IPF, shifting attention from nonspecific chronic inflammation toward organized, disease-associated macrophage programs (Fig. 3). Although alveolar macrophages are essential for tissue homeostasis, single-cell studies have identified distinct subpopulations that appear to contribute to fibrotic remodeling [86].

Two single-cell studies published in 2019 identified profibrotic alveolar macrophage populations in human IPF lungs [43, 44]. One study identified an IPF-enriched macrophage subset characterized by profibrotic mediators including *IL1RN, MMP9, CHI3L1, and SPP1* [44]. Another identified a similar SPP1^hi^ MERTK + population that markedly expanded in the lower lobes of fibrotic lungs, in contrast to the FABP4^hi^ macrophages that predominate in healthy alveolar spaces [43]. Although these initial reports used different nomenclature, they collectively suggested that the IPF microenvironment is characterized by the accumulation of macrophages with a distinct profibrotic transcriptional signature.

Subsequent single-cell analyses further consolidated this macrophage phenotype, showing that it is preferentially localized within distal fibrotic niches and characterized by programs extending beyond matrix-remodeling enzymes such as MMP9 and CTSK to include genes related to lipid handling and metabolic remodeling, such as LIPA, as well as niche-associated markers including GPC4, PALLD, CD84, and CD36 [32, 45]. Spatial transcriptomic analyses further support the close proximity of SPP1^hi^ macrophages to CTHRC1 + fibroblasts and aberrant epithelial populations within fibrotic regions, consistent with a multicellular niche that may reinforce fibroblast activation and impaired epithelial repair [87, 88]. Lineage-tracing and single-cell studies in murine models indicate that these cells primarily arise from recruited monocytes rather than resident macrophages [47, 89]. Following lung injury, monocyte-derived alveolar macrophages (Mo-AMs) transition through intermediate states marked by Cx3cr1, Ccr2, and Mafb before accumulating in fibrotic regions [46]. This profibrotic phenotype is maintained in part by local signaling within the niche, including M-CSF/M-CSFR interactions [47].

Together, single-cell studies identify SPP1^hi^ profibrotic macrophages as a key component of the IPF immune microenvironment. Rather than serving merely as bystanders of chronic inflammation, these macrophages participate in multicellular crosstalk with activated fibroblasts and injured epithelial cells through matrix remodeling, profibrotic inflammatory signaling, and persistence within distal fibrotic microenvironments (Fig. 3A).

#### Hypoxia-associated endothelial and vascular remodeling in IPF

The pulmonary vascular compartment is integral to alveolar gas exchange, endothelial barrier regulation, immune cell trafficking, and tissue repair. In IPF, vascular and endothelial abnormalities are increasingly recognized as part of the fibrotic remodeling process rather than merely secondary consequences of established scarring [90, 91]. Distortion of the distal lung architecture, remodeling of the alveolar-capillary interface, and loss or redistribution of the microvasculature can compromise oxygen diffusion, while localized hypoxia has been linked to profibrotic stress responses in injured lung tissue [92]. These changes link endothelial remodeling to impaired gas exchange, hypoxic stress, and fibrotic niche formation.

Single-cell studies have defined disease-associated endothelial states in IPF. A large-scale comparative scRNA-seq analysis of healthy, IPF, and COPD lungs identified ectopic COL15A1 + peribronchial endothelial cells, normally enriched around proximal airways, within fibrotic distal parenchyma and regions of pathological angiogenesis in IPF. The same study also associated IPF with venous remodeling near collagen-rich lesions and capillary loss in fibrotic foci, suggesting that vascular remodeling involves both aberrant vascular expansion and disruption of the normal alveolar-capillary network [32]. Human lung endothelial cell atlases have distinguished general capillary endothelial cells from alveolar capillary endothelial cells, providing a framework for interpreting capillary remodeling in diseased lung tissue [93]. In IPF, pathological remodeling of blood and lymphatic capillaries further supports the vascular component of the fibrotic niche [94].

At the molecular level, FOXF1 downregulation in IPF endothelial cells has been linked to fibrosis through the FOXF1-R-RAS axis, supporting endothelial contributions to tissue remodeling [95]. In aged bleomycin-injured lungs, Ackr1 + venous endothelial cells with inflammatory and angiogenic signatures accumulate, whereas persistent TrkB + gCap states suggest disrupted vascular homeostasis involving TrkB/YAP/TAZ signaling [48]. More recent single-cell and multi-omics studies have functionally implicated endothelial profibrotic pathways, including mechanosensitive PIEZO1-IL33 signaling linked to fibroblast activation [96] and RAP2A-MAP4K4-VCAM1 signaling associated with inflammatory endothelial activation and immune-vascular crosstalk [97]. Together, these data suggest that IPF endothelial remodeling contributes to vascular distortion and hypoxic, inflammatory, and profibrotic microenvironments that sustain fibrotic lesions (Fig. 4A).

#### Spatially organized fibrotic niches in IPF

While scRNA-seq defines epithelial, mesenchymal, and immune cell states associated with IPF, it does not determine how these populations are positioned within diseased tissue. Spatial transcriptomics provides a complementary layer by reconstructing fibrotic lesion architecture in situ and identifying multicellular niches that sustain aberrant repair (Fig. 3B).

Early spatial studies showed that fibrotic remodeling in the lung is regionally organized. A spatial human lung atlas described gland-associated immune niches and bronchial-associated fibroblast populations relevant to IPF [98]. Subsequent lineage tracing and single-molecule FISH localized distinct macrophage populations within fibrotic regions and implicated M-CSF/M-CSFR signaling in fibrotic niche maintenance [47]. Digital spatial profiling (GeoMx DSP) further identified senescence-associated programs, including p16-associated signals, enriched within fibroblast foci [99], and revealed region-specific epithelial signatures linked to NFKBIZ-dependent TGF-β/IL-6 crosstalk [100]. Other spatial studies identified GREM1-high fibroblasts as candidate mediators of remodeling [101], while murine Stereo-seq analyses resolved the dynamic redistribution of epithelial, mesenchymal, and immune states during fibrosis progression [102]. Together, these studies established the feasibility of mapping pathogenic microenvironments in fibrotic lung tissue, while highlighting the need for a more integrated view of human IPF architecture.

Recent comparative spatial atlases of human IPF and murine fibrosis have addressed this gap [87, 103]. In murine models, epithelial regeneration largely retains an AT2–ADI–AT1 trajectory consistent with ongoing alveolar repair [33–35, 103]. In contrast, human IPF is characterized by aberrant epithelial trajectories that divert into persistent KRT5-/KRT17 + basaloid cells rather than productive regeneration [28, 103]. These cross-species comparisons indicate that murine fibrosis does not fully recapitulate the stalled, non-regenerative spatial programs that define human IPF. Current ST data further show that these abnormal cell states are organized into distinct pathological niches rather than randomly dispersed throughout the lung [87]. Three major niche configurations have been described, including a fibrotic niche enriched for activated mesenchymal populations such as myofibroblasts and aberrant basaloid cells, an airway macrophage niche containing SPP1 + macrophages, and a lymphocytic immune niche [87]. Recent integrated spatial and single-cell analyses have further refined the fibrotic niche by distinguishing fibroblastic foci from dense fibrosis and identifying pathological fibroblast states enriched in these regions, including CTHRC1 + and WNT5A + CTHRC1 + myofibroblasts. PAK2-associated signaling further illustrates how spatially resolved fibroblast states may nominate pathways within active lesions [77].

Within fibrotic regions, spatial analyses further suggest that matrix-associated programs extend beyond collagen-rich fibrosis alone. In particular, ECM-dominant interaction networks involving collagen, laminin, fibronectin, and thrombospondin are enriched within the fibrotic niche, with myofibroblasts providing many of these signals and aberrant basaloid cells contributing ECM-related features such as FN1 and TNC expression [87]. Consistently, spatial factor analysis identified ECM- and fibrosis-related programs aligned with fibroblastic foci and associated with KRT5 −/KRT17 + aberrant basaloid cells, myofibroblasts, and HAS1^hi^ fibroblasts in human IPF tissue [103]. These observations indicate that fibrotic niches are organized by both local cellular composition and regionally patterned matrix signaling. High-resolution spatial and matrix-integrated analyses have further refined this view. In experimental fibrosis, distinct fibroblast neighborhoods were associated with ECM-secreting versus pro-resolving states, exemplified by Csmd1 + ECM-secreting fibroblasts enriched in fibrotic regions and Cd248 +/Pi16 + pro-resolving fibroblasts enriched in repair-associated areas [104]. Imaging-based transcriptomics has mapped molecular gradients across distal IPF lesions and linked epithelial dysregulation and macrophage polarization shifts to progressive architectural distortion [88]. Complementing this lesion-scale analysis, high-dimensional imaging of human IPF tissues across different stages of fibrotic remodeling identified a persistent spatial association between CD206^hi^ macrophages and alveolar-basal intermediate (ABIs) epithelial cells within the regenerating alveolar niche. Spatial receptor-ligand analysis further implicated fibronectin- and MIF-related signaling in this interaction, linking immune-epithelial crosstalk to abnormal alveolar regeneration in IPF [105]. Comparative spatial profiling across reversible and progressive fibrotic lung diseases further suggested that fibroblast fate is shaped by the surrounding microenvironmental niche. In this framework, IPF is associated with bronchiolized epithelial niches coupled to ECM-producing, apoptosis-resistant mesenchymal programs, whereas reversible lesions show spatial contexts more compatible with repair [106].

Taken together, spatial omics show that IPF is defined not only by aberrant epithelial, mesenchymal, and immune states, but also by their assembly into persistent pathogenic niches. This spatial framework provides an essential context for interpreting multicellular crosstalk in situ and may inform therapeutic strategies aimed at disrupting fibrosis at the level of tissue organization (Fig. 3B).

### COPD: compartment-resolved remodeling from small airways to emphysematous parenchyma

**Fig. 5:**
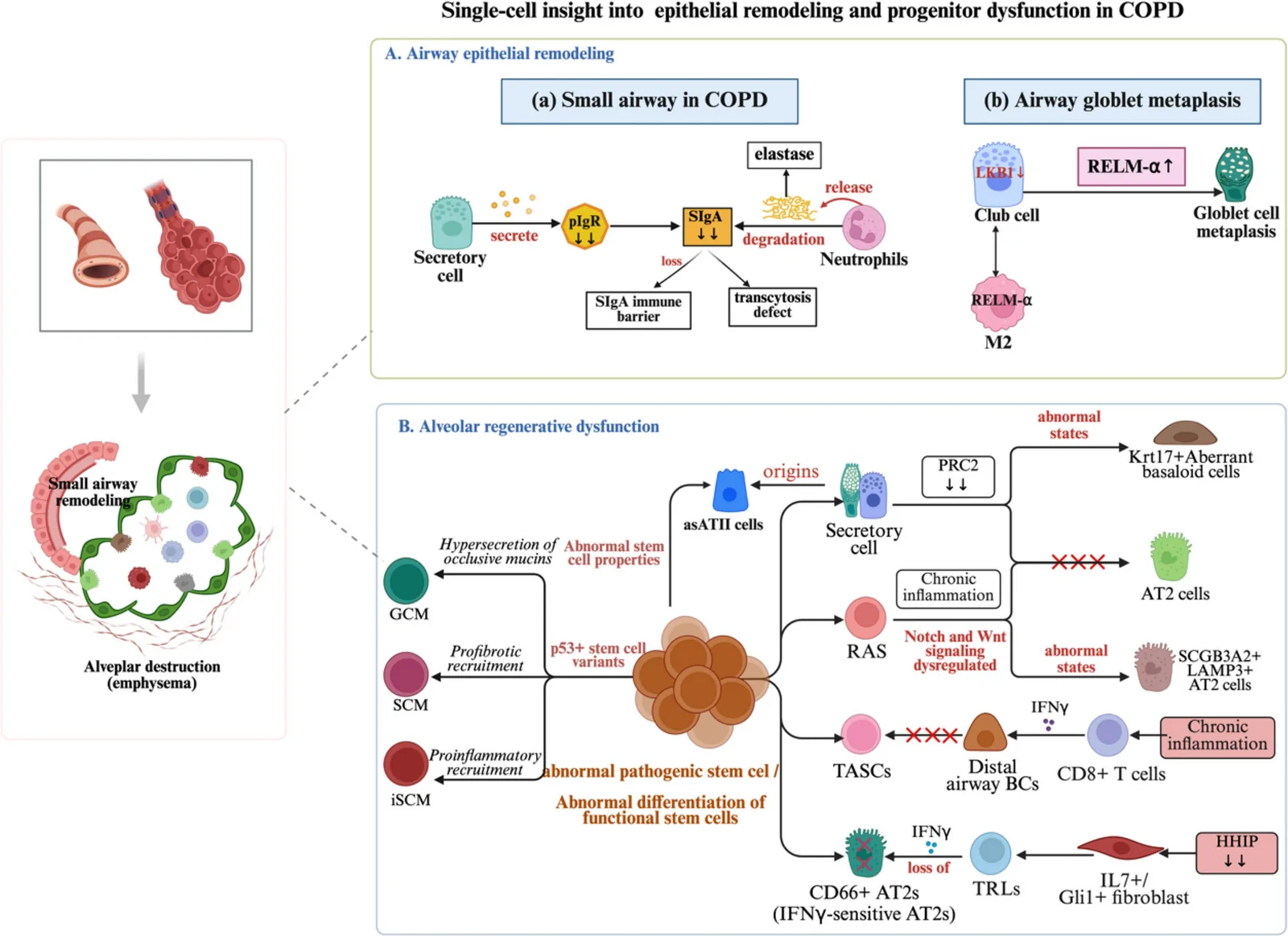
Single-cell transcriptomic insights into airway epithelial remodeling and alveolar regenerative dysfunction in COPD. **A** Airway epithelial remodeling in COPD, including small-airway epithelial alterations, barrier dysfunction, inflammatory responses, and goblet cell metaplasia. **B** Alveolar regenerative dysfunction in COPD, highlighting abnormal progenitor activation, pathogenic transitional cell states, impaired alveolar maintenance, and emphysematous destruction. Abbreviations: BCs, basal cells; RAS, respiratory airway secretory; TASCs, terminal airway-enriched secretory cells; GCM, goblet cell metaplasia; SCM, squamous cell metaplasia; iSCM, inflammatory SCM; TRLs, Tissue-resident lymphocytes; HHIP, hedgehog interacting protein; pIgR, Polymeric immunoglobulin receptor; SIgA, secretory IgA; LKB1, Liver kinase B1; RELM-α, Resistin-like molecule-α. Figure created in https://BioRender.com

**Fig. 6:**
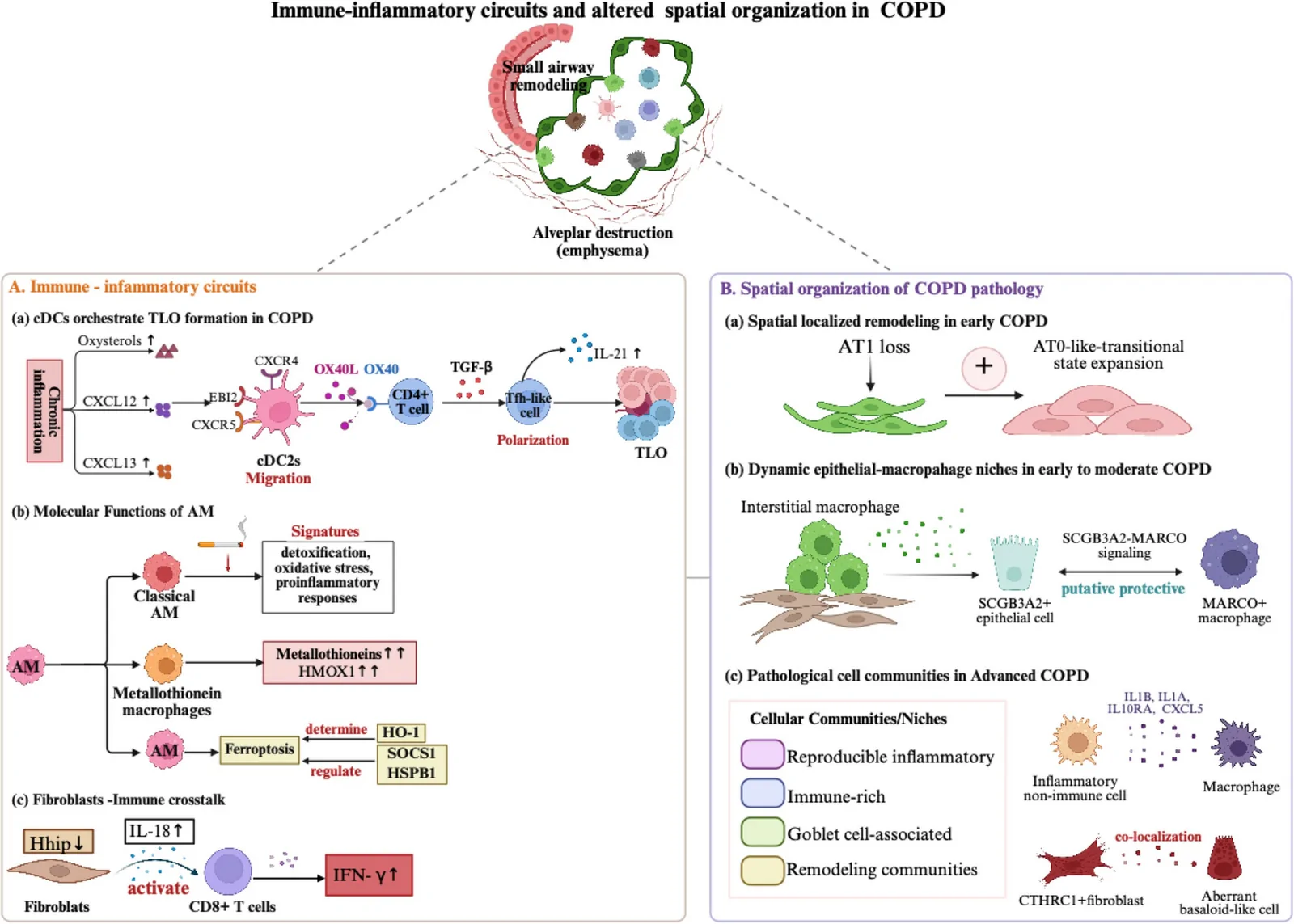
Single-cell and spatial transcriptomic insights into immune-inflammatory circuits and spatially organized pathology in COPD. **A** Immune-inflammatory circuits in COPD, including cDC-associated tertiary lymphoid organ formation, altered alveolar macrophage programs, and fibroblast–immune crosstalk. **B** Spatial organization of COPD pathology, including localized remodeling niches, epithelial–macrophage interactions, and pathological cellular communities identified by spatial transcriptomic analyses. Abbreviations: Tfh, T follicular helper; AM, alveolar macrophage; cDC, conventional dendritic cell; TLO, tertiary lymphoid organ; TRLs, tissue-resident lymphocytes; EBI2, Epstein-Barr virus-induced gene 2-positive. Figure created in https://BioRender.com

COPD is a heterogeneous and compartmentally organized disease in which small-airway remodeling and distal parenchyma destruction are closely linked yet distinct. Single-cell and spatial transcriptomic studies refine this framework by linking bronchiolar remodeling, emphysema, immune heterogeneity, endothelial stress, and spatial niche organization to impaired epithelial maintenance and persistent tissue injury (Table 2) (Figs. 4, 5 and 6).

#### Compartment-specific epithelial remodeling and progenitor dysfunction

Chronic exposure to cigarette smoke and other inhaled pollutants disrupts airway epithelial barrier integrity, promotes mucus hypersecretion, and alters the function of secretory and ciliated cell populations, contributing to airway narrowing and impaired mucociliary clearance [107, 108]. Single-cell analysis showed that cigarette smoke alters secretory and ciliated epithelial cell states in COPD airways [109]. Club cell dysfunction, including loss of LKB1, has been linked to defective epithelial differentiation and M2-like macrophage polarization after smoke exposure [110]. Beyond mucus-related remodeling, impaired mucosal immune defense is also evident in COPD airways, as secretory IgA deficiency has been linked to small-airway pathology [111]. Single-cell analysis identified secretory cells as the major source of pIgR, which mediates IgA transcytosis, and suggested that impaired SIgA transport in COPD reflects reduced pIgR expression or protein rather than a proportional loss of secretory cells [112] (Fig. 5A).

These abnormalities reflect progenitor dysfunction rather than terminal differentiation changes alone. Single-cell cloning analyses identified metaplastic stem/progenitor populations associated with goblet cell metaplasia (GCM), squamous cell metaplasia (SCM), and ‘inflammatory SCM’ (iSCM), suggesting that chronic injury reshapes the progenitor pool [54]. In healthy distal airways, basal cells (BCs) serve as multipotent progenitors [61], and single-cell atlases have described a basal cell continuum spanning healthy and COPD lungs [113]. Human distal airway studies have shown that smoking and COPD are associated with distal-to-proximal repatterning of the small airway epithelium, a process linked to altered basal cell differentiation [114]. Subsequent single-cell analysis identified terminal airway-enriched secretory cells (TASCs) as a distal secretory population enriched in terminal bronchioles (TBs) and showed that loss of pre-TBs and TBs in COPD is accompanied by impaired epithelial regenerative capacity in this compartment [52], likely reflecting disease-associated intrinsic changes in BCs. This aligns with evidence that airway basal cells self-renew and generate major airway epithelial lineages, supporting their role as critical progenitor sources [115]. Epigenetic dysregulation contributes to this defect. PRC2/EZH2-dependent programs are required for basal stem cell self-renewal and orderly differentiation, whereas their disruption promotes abnormal KRT17 + basal-like states associated with squamous reprogramming [116–118].

Progenitor dysfunction also extends to the bronchioalveolar junction. Respiratory airway secretory (RAS) cells have been proposed as AT2 progenitors, and transcriptomic analyses indicate that this differentiation axis is perturbed in COPD [49]. Murine transplantation studies further show that distal airway stem/progenitor cells (DASCs) can generate Krt5 + Ki67 + progenitor-like cells and differentiate into Hopx + Aqp5 + AT1 cells in vivo, supporting a potential role for distal airway progenitors in alveolar repair [53]. Thus, small-airway remodeling in COPD reflects secretory and ciliated cell dysfunction together with failure of basal and distal airway progenitor programs that maintain epithelial integrity and shape repair trajectories [119] (Fig. 5B). Compared with IPF, where epithelial dysfunction centers on AT2 failure, persistent transitional states, and fibrotic alveolar remodeling, COPD more prominently involves compartment-specific airway epithelial dysregulation and impaired maintenance of the distal gas-exchange unit.

#### Distal parenchymal injury and macrophage-associated alterations in emphysematous lungs

In the distal lung, emphysematous destruction is associated with abnormal alveolar epithelial states marked by stress susceptibility, altered lineage identity, and impaired regeneration. Sauler et al. identified an AT2 subpopulation in emphysematous COPD lungs, termed AT2B cells, characterized by metabolic dysregulation, reduced stress tolerance, and HHIP-linked susceptibility to cigarette smoke-induced injury, and further linked reduced NUPR1 expression to increased vulnerability of AT2 cells to ferroptotic death [29]. Additional work identified inflammatory AT2 states in smokers, marked by chemokines such as CXCL1 and CXCL8, consistent with a proinflammatory epithelial response to smoke exposure [120]. Single-cell analyses of emphysematous distal lung regions further identified disease-specific SFTPC + SCGB3A2 + asATII cells within alveolar sacs, which co-express alveolar and club-associated markers and show features of aberrant progenitor activation, including senescence, mitochondrial dysfunction, inflammatory signaling, apoptosis-related programs, WNT suppression, and impaired regenerative potential [51]. Additional genetic evidence showed that cigarette smoke-induced reduction of epithelial *TACC2* promotes alveolar inflammation, epithelial cell death, and emphysematous change [121]. Together with impaired RAS-to-AT2 differentiation, these stress-associated epithelial states support the view that emphysema reflects weakened renewal at the bronchioalveolar interface rather than alveolar wall loss alone [49].

Immune remodeling in emphysematous lungs is strongly shaped by macrophage heterogeneity. Single-cell RNA sequencing identified both resident smoke-responsive alveolar macrophages and expanded monocyte-derived macrophage populations expressing COPD-associated programs such as CLEC5A and VCAN [122]. These data indicate that smoke exposure shifts the alveolar macrophage compartment toward multiple inflammatory and injury-associated states rather than activating a single uniform response. Macrophage dysregulation in COPD is also linked to altered iron homeostasis [123], lipid remodeling [124], and heme metabolism [125]. Smokers and patients with COPD exhibit increased iron burden in bronchoalveolar lavage [126], while advanced COPD is associated with metallothionein-high alveolar macrophage states [29]. Other studies linked macrophage ferroptosis-related programs and HO-1-centered stress responses to COPD severity [127, 128]. Notably, recent analyses of mild-to-moderate COPD identified an interstitial macrophage population with a comparatively anti-inflammatory transcriptional profile, suggesting that macrophage remodeling may be stage-dependent and may include transient compensatory states before more overt inflammatory programs predominate [129]. In advanced COPD, a multimodal single-cell study identified expansion of a CHIT1-high interstitial macrophage state. These profibrotic IM_CHIT1_ subset expressed matrix-remodeling genes such as CHI3L1, MMP9, and SPP1 and were reported to contribute in part to the adverse association between smoking exposure and lung function decline [55]. In contrast to the SPP1^hi^ profibrotic macrophage niche in IPF, macrophage remodeling in COPD appears more stage-dependent and metabolically heterogeneous.

Single-cell data also point to broader immune-stromal circuits that amplify distal lung destruction. Monocyte-associated programs have been linked to alveolar epithelial apoptosis and autoimmune-like responses in COPD [130]. In murine emphysema models, reduced HHIP activity in fibroblasts promotes IL-7- or IL-18-associated stromal signaling, expands tissue-resident lymphocyte populations, and impairs epithelial stem cell renewal [50, 131]. Related work suggested that dysregulated Gli1 + fibroblasts can drive depletion of a clonogenic, IFNγ-sensitive CD66c + AT2 stem cell subpopulation through tissue-resident lymphocyte-dependent mechanisms, thereby exacerbating emphysematous injury [50]. In advanced human disease, this remodeling is accompanied by tertiary lymphoid organization, with cDC2 populations promoting Tfh-like polarization and local lymphoid neogenesis [132, 133]. A recent smaller-scale single-cell study further reported progressive depletion of a metallothionein-high T-cell subset from healthy controls to pre-COPD and established COPD samples, suggesting loss of an immunoregulatory state that may normally restrain CD8 + T-cell cytotoxicity and potentially serve as an early susceptibility marker [134]. Overall, distal COPD pathology is better understood as a coordinated failure of epithelial maintenance coupled to macrophage-associated, stromal, and immune remodeling rather than alveolar wall loss alone (Fig. 6A).

#### Endothelial stress, hypoxic stress, and vascular remodeling in COPD

In COPD, small-airway obstruction, air trapping, emphysematous alveolar destruction, and ventilation-perfusion mismatch disrupt the alveolar-capillary unit, reduce gas-exchange surface area, and expose distal lung regions to hypoxic stress [135]. These abnormalities disrupt the alveolar-capillary unit, reduce the effective gas-exchange surface, and expose distal lung regions to hypoxic stress. Pulmonary vascular dysfunction and endothelial remodeling are therefore relevant to COPD pathobiology, particularly in emphysematous compartments [136].

Integrated single-cell and spatially resolved studies now provide a more detailed view of this vascular component. Earlier single-cell analyses showed inflammatory activation of COPD capillary endothelial cells, with prominent gCap transcriptomic alterations in advanced disease, and network analyses implicated CXCL-associated signaling, including CXCL12, as a contributor to COPD pathogenesis [29]. These disease-associated capillary endothelial programs included cellular stress and inflammatory features, with increased expression of TNFRSF10D and IRF1 in aerocytes and increased expression of TNFAIP3, IFI6, and IL6 in gCaps in advanced COPD [29]. More recent large-scale snRNA-seq integrated with spatial transcriptomics and proteomics linked shifts in endothelial and other non-immune cell states to lung function decline, emphysema, and spatially organized COPD niches [55]. Within this framework, inflammatory gCap activation, loss of gas-exchange-associated aerocytes, and reduction of reparative endothelial programs suggest that COPD vascular remodeling reflects both inflammatory stress and impaired repair of the alveolar-capillary interface [55]. Integrated proteomic, bulk transcriptomic, single-cell, and spatial analyses suggest that endothelial cells in severe COPD acquire senescence-associated programs with elevated IL6 expression, linking endothelial stress to altered T-cell activity [56]. This pattern differs from IPF, where endothelial remodeling is embedded within collagen-rich fibrotic niches and shaped by abnormal angiogenic, mechanosensitive, and immune-vascular signaling. In COPD, vascular changes are more closely aligned with emphysematous loss of the gas-exchange unit, inflammatory capillary endothelial states, impaired endothelial repair, and airway-parenchymal heterogeneity (Fig. 4B).

#### Spatial organization of COPD pathology

Compared with IPF, spatially resolved studies in COPD remain relatively limited, but they are beginning to show how disease-related cell states are organized across the small airway- alveolar axis. This is particularly relevant in COPD, where airflow limitation, bronchiolar remodeling, and emphysematous destruction do not occur uniformly throughout the lung. Recent spatial analyses of healthy and diseased human lung tissue further show that epithelial composition, immune cell distribution, and stromal organization vary markedly by region and are altered even in early-stage COPD [137]. By establishing a spatial reference map of healthy adult lung and applying it to GOLD II COPD, the study showed that early disease is characterized not by diffuse epithelial disruption, but by localized remodeling near alveolar regions and respiratory bronchioles, where AT1 loss and expansion of AT0-like transitional epithelial states were linked to distinct tissue neighborhoods, including AT0-enriched alveolar regions, immune cell-enriched parenchyma, and depleted alveolar capillary niches [137]. These findings indicate that abnormal epithelial differentiation, inflammatory infiltration, and structural simplification converge within specific anatomical regions rather than occurring uniformly across the lung.

Early spatial niches in COPD may not all be deleterious. In mild-to-moderate COPD, spatially informed single-cell analysis identified an interstitial macrophage niche with an anti-inflammatory profile, together with a putative SCGB3A2-MARCO epithelial-macrophage configuration consistent with local homeostatic support [129]. These observations suggest that some epithelial-macrophage niches may initially buffer tissue injury or help preserve local homeostasis. As disease progresses, however, spatial studies have highlighted immune-rich microenvironments associated with persistent inflammation and remodeling. B cell-rich lymphoid follicles have long been associated with emphysema severity [138, 139], and digital spatial profiling has confirmed that these structures are regionally enriched in COPD lungs and display distinct transcriptional programs [140]. In severe COPD, pathological cell states are not randomly distributed but assemble into reproducible inflammatory, immune-rich, goblet cell-associated, and remodeling communities whose distribution correlates with lung function, symptom burden, and exacerbation-related features [55]. Within these communities, spatial co-localization of CTHRC1 + fibroblasts with aberrant basaloid-like cells, together with the proximity of inflammatory non-immune cells to macrophages expressing IL1B, IL1A, IL10RA, and CXCL5, provides an anatomical basis for signaling networks that may sustain chronic inflammation and tissue remodeling [55]. Together, these findings support a view of COPD as a spatially heterogeneous disease in which clinical variability reflects the **r**egional arrangement and interaction of pathological niches rather than uniform tissue injury (Fig. 6B). However, interpretation of these spatial patterns remains constrained by sampling bias, clinical heterogeneity, and limited access to early-stage human tissue.

### Translational implications, current limitations, and future directions

The single-cell and spatial studies discussed above have not yet yielded a clinically deployable precision-medicine framework for IPF or COPD. Nevertheless, they have reshaped how translational opportunities are prioritized (Table 3). By resolving these diseases into cell-state-specific and niche-dependent pathological programs, these approaches help identify processes linked to disease progression, tissue remodeling, and therapeutic resistance. At present, their most immediate translational value lies in refining molecular stratification, prioritizing targets within disease-relevant cellular programs and niches, and guiding the selection of experimental systems for functional validation.

**Table 3:** Target prioritization and mechanism-based interventions in IPF and COPD

Disease	Targets/Candidate Drug	Target Population/Pathway	Intervention Strategy	Targeted Outcomes	Ref
IPF	CCL18	IPFeMΦ; CCL18/CCL22	Small molecule: CRT and Cucur	Reverse the signature of IPF-specific macrophages; Reduce CCL18 production	[45]
IPF	M-CSF/M-CSFR	Monocyte-derived alveolar macrophages	Pharmacological blockade of M-CSFR signalling (anti-CSF1 antibody, PLX3397)	Disrupt the fibrotic niche and ameliorates fibrosis	[47]
IPF	IgA secretion	AT2-like epithelial cells; fibroblasts	Targeted blockade of IgA secretion by pulmonary epithelial cells	Reduce IgA deposition in ECM; Inhibit fibroblast activation; Ameliorate pulmonary fibrosis	[141]
IPF	Notch signaling	AF1-derived AF2 cells; Krt5 + basal cells	Inhibition of intracellular Notch signaling in AF1 cells	Block injury-induced niche formation; Rewire signaling to favor euplastic regeneration; Improve lung function	[42]
IPF	CXCL6	CXCL6 in abnormal Secretory cells; CXCR1/2 signaling pathway in fibroblasts	Chemically inhibit CXCR1/2 with reparixin; Silence CXCR1/2 gene	Block CXCL6-induced collagen synthesis in fibroblasts	[142]
IPF	TWIST1	Myofibroblasts, E-box transcription factor motifs	Overexpress Twist1 in Col1a2-expressing fibroblasts	Increase collagen synthesis and α-SMA expression; Identify TWIST1 as critical regulator of myofibroblast activity	[85]
IPF	GPR87	Distal bronchiolar KRT5 + basal-like cells; epithelial differentiation pathways	Overexpress GPR87 in primary human bronchial epithelial cells (HBECs)	Impair airway cell differentiation, reduce cilia coverage; Identify GPR87 as a basal cell marker in honeycomb cysts	[143]
IPF	PPP2R3A	Inflammatory-proliferative fibroblasts (gremlin1^hi^ fibroblasts)	Knocking down PPP2R3A expression	Block early pathological changes in IPF	[101]
IPF	Nintedanib	Activated fibroblasts, Receptor tyrosine kinases (RTKs)	RTK inhibition; Reduces ECM-associated transcriptional programs	Inhibits the fibrotic process and extracellular matrix expansion	[74]
IPF	Saracatinib	Aberrant basaloid cells (ABCs), SRC kinase pathway	Pharmacological inhibition of SRC kinase	Reduces bronchosphere formation; Blocks SRC-mediated pro-fibrotic signaling	[65]
IPF	Senolytic drugs (dasatinib + quercetin)	Senescent AT2 cells	Targeted elimination of senescent AT2 cells (dasatinib + quercetin)	Reduces progressive alveolar senescence and fibrotic remodeling	[144]
IPF	Pifithrin-α (p53 inhibitor)	Senescent AT2 cells; p53 signaling pathway	Pharmacological inhibition of p53 activation	Inhibits p53 activation; Reduces AT2 cell senescence and fibrosis	[144]
IPF	FOXF1/R-Ras axis	Endothelial cells; FOXF1-R-Ras signaling pathway	Endothelial FOXF1 restoration or endothelial-specific FOXF1 gene delivery	Reduce collagen deposition, inflammation, and fibrosis; improve lung function	[95]
COPD	HHIP	Peribronchial fibroblasts; HHIP-Hedgehog axis	Stromal-specific HHIP restoration	Attenuate TRL niche and TRL expansion; Improve alveolar regeneration capacity	[50]
COPD	DASC therapy	Distal airway stem cells	Transplant DASC	Repair lung tissue damage; Improve pulmonary function	[53]

#### Translational opportunities and target prioritization

Single-cell and spatial transcriptomic studies are shifting therapeutic discovery from broad pathway inhibition toward more selective targeting of disease-relevant cell states and tissue niches. In IPF, this approach has been especially informative because several candidate targets have been supported by independent single-cell, spatial, and functional studies. Integrated analyses showed that IgA produced by alveolar epithelial cells can accumulate within fibrotic extracellular matrix and directly activate fibroblasts [141], supporting an epithelial-associated profibrotic program that may contribute to biologic heterogeneity in IPF [145]. Similarly, the recurrent identification of CTHRC1 + fibroblasts as a central fibrogenic population has focused attention on a fibrogenic population that is transcriptionally activated and spatially localized within active remodeling lesions [40]. At the epithelial–stromal interface, the balance of Notch, Wnt, and Fgf signaling appears to influence whether injured alveolar epithelium undergoes regenerative repair or persistent fibrosis, and experimental evidence suggests that modulating this balance, including Notch inhibition in AF1 cells, may reduce aberrant repair and promote alveolar regeneration [42]. Spatial transcriptomic studies extend this framework by defining fibrotic and immune niches whose molecular composition may help stratify IPF lesions and progression patterns [87, 88, 103].

Transcriptomic atlases also help link abnormal cell states to clinical trajectories. Human alveolar epithelial type 2 cells can adopt metaplastic basal-like programs in fibrotic settings, and the abundance of these KRT5 + epithelial cells correlates with adverse clinical outcomes [31, 146]. Single-cell eQTL analyses have further highlighted context-specific genetic regulation, linking disease-associated variants to discrete cell populations rather than to bulk lung tissue [147]. Cell-state-resolved studies have also revealed candidate therapeutic entry points, including CXCL6-CXCR2 signaling, which promotes fibroblast collagen synthesis [142], TWIST1-dependent myofibroblast programs [85], and GPR87-associated dysregulation of basal-like epithelial differentiation [143]. Niche-associated targets such as PPP2R3A in inflammatory fibroblasts [101] and M-CSFR in alveolar macrophages [47, 101] further illustrate how integrated omics can prioritize intervention points within active fibrotic circuitry. Endothelial-directed strategies have also been proposed, as restoration of FOXF1-R-Ras signaling in lung endothelial cells reduced collagen deposition, inflammation, and fibrosis while improving lung function in experimental models [95]. Beyond cell-associated targets, extracellular niche signals such as CCL18 and CCL7 provide a rationale for exploring biologic therapies in IPF [148]. Consistent with this, signature-based drug repurposing approaches have identified candidate compounds that suppress CCL18-associated macrophage programs [45]. Such extracellular or surface-accessible targets may therefore be more tractable for biologic intervention than intracellular transcriptional programs. Overall, these studies help distinguish broadly dysregulated pathways from those embedded in active fibrotic circuitry, although whether niche-specific ligand-receptor targeting provides greater cellular selectivity than systemic pathway inhibition remains to be tested.

Compared with IPF, translational prioritization in COPD is more strongly shaped by anatomical and phenotypic heterogeneity. Nevertheless, useful stratification is beginning to emerge by focusing on compartment-specific repair failure rather than undifferentiated inflammation alone. Disruption of progenitor-linked programs in basal cells, distal airway stem cells, and the respiratory airway secretory (RAS)-to-AT2 axis [49], together with the emergence of aberrant asATII states [51] and epithelial-specific effects of TACC2 perturbation [121], suggests that impaired epithelial renewal may represent an important therapeutic target class in COPD. Single-cell studies have also implicated an HHIP-regulated tissue stromal-immune axis, supporting the idea that loss of regenerative stromal support may contribute directly to disease progression rather than simply reflecting late parenchymal destruction [50]. These findings suggest that therapeutic prioritization in COPD will likely need to distinguish airway-predominant remodeling, bronchioalveolar repair failure, and distal parenchymal destruction rather than assuming a single shared pathogenic target. At present, however, these observations are more useful for mechanism-guided stratification than for defining immediately deployable targeted therapies (Table 3).

Overall, current translational opportunities are strongest when an implicated pathway is linked to a discrete disease-relevant cell state, supported across multiple datasets or modalities, and connected to a plausible intervention strategy. By this standard, single-cell and spatial studies have already sharpened target prioritization in IPF and have begun to provide a similar framework for COPD, although patient-level molecular stratification remains an emerging goal rather than an established clinical reality.

#### Functional validation and advanced experimental models

A major challenge in translating omics observations into therapy is that transcriptional correlation does not establish functional causality. The next step therefore requires experimental systems to test whether candidate cell states, signaling pathways, and intercellular interactions are causal, reversible, and pharmacologically tractable. Omics studies are most informative when paired with perturbation-based validation instead of being treated primarily as descriptive endpoints.

In IPF, single-cell studies of treatment response have begun to clarify drug mechanisms beyond bulk tissue readouts. For example, profiling studies showed that nintedanib reduces activated fibroblast populations and suppresses extracellular matrix-associated transcriptional programs [74], aligning with its preclinical and clinical efficacy [149]. Other work identified senescent AT2 cells as a therapeutically relevant epithelial state and supported senolytic strategies such as dasatinib plus quercetin to eliminate these dysfunctional cells [144]. In addition, scRNA-seq linked elevated SRC kinase activity in aberrant basaloid cells to fibrotic progression, and subsequent inhibition with saracatinib attenuated fibrosis in murine models [65]. These examples illustrate the broader value of omics-guided validation when a candidate target can be assigned to a defined pathogenic cell state and then perturbed experimentally.

In COPD, functional validation has more often centered on epithelial plasticity, barrier dysfunction, and regenerative failure. Experimental studies of distal airway stem or progenitor populations support the view that defective distal airway repair is mechanistically important and may represent a tractable therapeutic process [51]. Related work showed that transplanted distal airway stem cells can regenerate functional epithelium in murine injury models, supporting the feasibility of repair-oriented strategies in selected COPD-relevant contexts [53]. More broadly, pathways implicated by single-cell studies, including HHIP-linked stromal-immune regulation [50] and progenitor-associated Wnt abnormalities [51], point toward repair-oriented intervention strategies that differ conceptually from conventional anti-inflammatory approaches. However, these interpretations remain early and require more direct validation in human-relevant systems.

Advanced in vitro and ex vivo models are therefore important for functional validation. Human lung organoids are increasingly used to test epithelial lineage behavior, injury responses, and patient-specific perturbations, particularly when coupled with single-cell transcriptomic readouts that resolve disease-associated differentiation trajectories [150, 151]. In COPD, organoid systems combined with single-cell profiling have recapitulated altered epithelial differentiation trajectories and heightened inflammatory responses at the individual level [152]. However, organoids alone are not sufficient. Air–liquid interface cultures complement organoids by modeling airway differentiation, barrier dysfunction, mucociliary abnormalities, and cigarette smoke responses relevant to COPD [153, 154]. Precision-cut lung slices preserve native tissue architecture and multicellular composition more effectively than dissociated cultures, making them suitable for short-term interrogation of drug responses, fibrotic remodeling, and local cell–cell interactions in human lung tissue [155, 156]. Emerging organ-on-chip and mechanically active lung models further expand this toolkit by incorporating biomechanical stress, which is relevant to fibrosis and epithelial injury [157, 158]. A productive translational workflow will likely combine discovery in human single-cell and spatial atlases with iterative validation across complementary experimental platforms rather than relying on any single model.Together, these models are essential for determining whether omics-derived cell states and pathways are causal, reversible, and therapeutically tractable.

#### Current limitations and future directions

Despite major progress, several limitations still constrain the translation of single-cell and spatial findings into robust mechanistic models and therapeutic strategies. One persistent limitation is inconsistency in cell-state annotation, as overlapping populations may receive different names whereas distinct states may be grouped under the same label. This problem is evident for epithelial and immune states such as profibrotic macrophages [43–45, 87] and transitional epithelial populations [33–36], underscoring the need for transcriptomic, spatial, proteomic, epigenetic, and functional integration.

A second major limitation concerns the nature of available human tissue. Many lung omics studies still rely heavily on end-stage explants, lung volume reduction surgery samples, or histologically normal-appearing tissue adjacent to tumor resections. These materials are valuable, but they bias analysis toward advanced remodeling and complicate inference about the earliest events that initiate disease. In COPD, early airway-predominant disease and terminal bronchiolar loss are especially difficult to capture consistently in human cohorts [159]. Similar challenges apply to IPF, where transplantation or biopsy specimens often represent established disease and may not fully resolve the temporal sequence linking epithelial injury, immune activation, fibroblast expansion, and matrix remodeling. Moving from descriptive atlases to more faithful models of disease progression will require severity-resolved, regionally annotated, and longitudinal human sampling, ideally linked to imaging, physiology, and detailed clinical metadata. Multicenter sampling strategies and less invasive approaches, including cryobiopsy in appropriate settings, may help address this gap [160].

Experimental validation is further constrained by the limitations of animal models. Murine systems remain highly informative, but they do not fully reproduce the defining pathological architecture of human chronic lung diseases. In IPF, the standard bleomycin injury model is valuable for studying acute fibrogenic responses, but it does not recapitulate the patchy, progressive peripheral fibrosis and characteristic fibroblastic foci of human usual interstitial pneumonia [161, 162]. In COPD, cigarette smoke and elastase models capture selected inflammatory and destructive components of disease but do not adequately reproduce the coupled progression from small airway remodeling to distal parenchymal destruction that defines human COPD [163]. This discrepancy partly reflects anatomical differences, as the mouse lung lacks the complex terminal and respiratory bronchiolar branching network central to human small-airway pathology in COPD [49, 164]. These differences do not negate the value of animal models but they indicate that findings derived from them should be interpreted as partial mechanistic approximations rather than direct replicas of human disease.

Technical constraints also shape the interpretation of single-cell and spatial studies. Major trade-offs remain among transcriptome depth, single-cell or subcellular resolution, tissue coverage, histological fidelity, and the ability to capture extracellular matrix organization in the same experiment. Emerging high-resolution platforms, including imaging-based spatial methods, may help narrow these gaps, although their broader application remains limited by cost, throughput, and technical complexity [165, 166]. These issues are particularly relevant to chronic lung diseases, in which matrix organization, structural remodeling, and cell localization are inseparable aspects of pathogenesis [167–169]. Future progress will therefore depend on improved spatial transcriptomics and tighter integration with histology, proteomics, and extracellular matrix mapping. Broader sharing of raw data, analytical pipelines, and harmonized annotations, together with computational frameworks for cross-platform integration and biomarker prioritization, should improve reproducibility and support more reliable cross-cohort comparisons.

Together, these limitations highlight the need for stage-resolved human sampling, standardized cell-state definitions, and validation across complementary experimental platforms.

## Conclusion

Single-cell and spatial transcriptomic studies have advanced current understanding of IPF and COPD by resolving cellular heterogeneity, tissue niches, and intercellular interactions beyond the limits of conventional histology. Across both diseases, these approaches have helped shift interpretation of pathology from static descriptions toward dynamic, spatially organized multicellular processes. They also highlight shared pathological features, including epithelial injury, maladaptive repair, multicellular remodeling, and impaired gas exchange, while distinguishing the fibrotic niche formation that is more prominent in IPF from the airway-centered and emphysematous remodeling that characterizes COPD. At present, their value lies primarily in refining disease mechanisms rather than delivering immediate precision medicine. Future progress will depend on improved cross-study standardization, stage-resolved human samples, multimodal data integration, and rigorous functional validation. With these advances, single-cell and spatial omics may gradually contribute to a more testable and therapeutically informative framework for chronic lung diseases.

## Data Availability

No datasets were generated or analysed during the current study.

## Abbreviations

COPD: Chronic obstructive pulmonary disease
IPF: Idiopathic pulmonary fibrosis
SIgA: Secretory IgA
pIgR: Polymeric immunoglobulin receptor
LKB1: Liver kinase B1
RELM-α: Resistin-like molecule-α
ECM: Extracellular matrix
RAS: Respiratory airway secretory
TASCs: Terminal airway-enriched secretory cells
TBs: Terminal bronchioles
DASCs: Distal airway stem cells
GCM: Goblet cell metaplasia
SCM: Squamous cell metaplasia
iSCM: Inflammatory SCM
SPB: Secretory primed basal
ABIs: Alveolar-basal intermediates
LNEPs: Club-like lineage-negative epithelial progenitors
ADI: Krt8 + alveolar differentiation intermediate
DATPs: Damage-associated transient progenitors
PATS: Pre-alveolar transitional state
AF1/2: Alveolar Fibroblasts1/2
High-MT macrophages: High metallothionein macrophages
IPFeMΦ: IPF-expanded macrophages
AT1: Alveolar epithelial type 1
AT2: Alveolar epithelial type 2
MPB: Multipotent basal
AB: Activated basal
PB: Proliferating basal
ABCs: Airway basal cells
EVs: Extracellular vesicles
HHIP: Hedgehog interacting protein
EBI2: Epstein-Barr virus-induced gene 2-positive
TLO: Tertiary lymphoid organs
Tfh: T follicular helper
HO-1: Heme oxygenase-1
TRLs: Tissue-resident lymphocytes
BASCs: Bronchioalveolar stem cells
